# High Fermentable Oligosaccharides, Disaccharides, Monosaccharides, and Polyols (FODMAP) Consumption Among Endurance Athletes and Relationship to Gastrointestinal Symptoms

**DOI:** 10.3389/fnut.2021.637160

**Published:** 2021-04-20

**Authors:** Lauren A. Killian, Jane G. Muir, Jacqueline S. Barrett, Nicholas A. Burd, Soo-Yeun Lee

**Affiliations:** ^1^Division of Nutritional Sciences, University of Illinois at Urbana-Champaign, Urbana, IL, United States; ^2^Department of Gastroenterology, Central Clinical School, Monash University, Melbourne, VIC, Australia; ^3^Department of Kinesiology and Community Health, University of Illinois at Urbana-Champaign, Urbana, IL, United States; ^4^Department of Food Science and Human Nutrition, University of Illinois at Urbana-Champaign, Urbana, IL, United States

**Keywords:** endurance, exercise, FODMAP, irritable bowel syndrome, nutrition

## Abstract

Endurance athletes commonly experience lower gastrointestinal (GI) symptoms similar to those of irritable bowel syndrome (IBS). Previous research on the restriction of fermentable oligosaccharides, disaccharides, monosaccharides, and polyols (FODMAP), a diet-based mitigation strategy initially developed for IBS, has shown promise for application in athlete populations. Athlete's dietary strategies surrounding exercise have not been formally assessed in relation to FODMAP content of foods or sports nutrition products. Additionally, the FODMAP content of athlete's habitual diets has not been examined in larger sample sizes. This research aims to investigate the FODMAP content of endurance athlete diets by examining these three areas, in conjunction with GI symptoms. Dietary habits surrounding exercise and GI symptoms were examined in 430 endurance athletes using a previously validated Endurance Athlete Questionnaire. A subset of athletes (*n* = 73) completed a FODMAP-specific food frequency questionnaire for habitual intake. The most commonly reported sports nutrition products were analyzed for FODMAP content using standardized analytical methods. Mean habitual intakes were compared to previous FODMAP studies and medians were compared between those with and without lower GI symptoms. Athletes commonly consumed high FODMAP foods during pre-race dinners and breakfasts, with over 60% reporting specific high FODMAP foods. More frequent nutrition product use, particularly solid, gel/gummy, and homemade products, was often related to increased frequency of GI symptoms. Of the sixteen commonly used sports nutrition products tested, seven were high FODMAP in one serving. All but one of the remaining products became high FODMAP when consumed in multiple servings, as is likely the case during endurance exercise. Average habitual FODMAP intake was 26.1 g (±15.9 g), similar to intakes classified as high FODMAP in previous research on FODMAPs and IBS or GI symptoms. Only 15.1% of athletes consumed a diet that would be considered low in FODMAP. Exploratory analyses showed higher intake of some FODMAP types among athletes exhibiting various lower GI symptoms. Overall, this study demonstrated that FODMAP intake by endurance athletes is high both surrounding exercise and habitually, and may be contributing to GI symptoms experienced during exercise. This information can be utilized when analyzing athlete diets and selecting foods to decrease GI symptoms.

## Introduction

Lower gastrointestinal (GI) symptoms are common among endurance athletes, can interrupt or prevent training and competition, and have the potential to impact performance (1–6). The symptoms experienced by athletes, such as GI cramps/pain, bloating, and diarrhea, are similar to those of patients with irritable bowel syndrome (IBS), a functional GI disorder characterized by abdominal pain and altered bowel habits that affects 10–20% of the U.S. population (7–11). In a recent assessment of marathon, ultramarathon, half distance, or full distance triathlon athletes, 9.8% had either been diagnosed with IBS or met Rome III IBS diagnostic criteria (4). Additional athletes reported experiencing IBS-like symptoms despite not meeting IBS criteria.

Recent studies in patients with IBS have shown success with dietary behavioral interventions; specifically limiting specific short-chain carbohydrates known as fermentable oligosaccharides, disaccharides, monosaccharides, and polyols (FODMAP) (12–14). These carbohydrates have been shown to be poorly absorbed, osmotically active, and highly fermentable, which contributes to the GI symptoms experienced by those with IBS (14–16). Restriction of FODMAPs has been shown to improve symptoms in ~70% of patients with IBS, demonstrating the contribution of nutritional components to lower GI symptom genesis (14, 17, 18).

It is hypothesized that GI symptoms of athletes result from a combination of issues stemming from mechanical, psychological, physiological, and nutritional causes (3, 19–21). Relatively recent reviews such as that by Costa et al. (19) provide a comprehensive understanding of all factors involved. When an athlete exercises at the intensity and for the duration characteristic of endurance activities, most of these factors are not easily modifiable. Athletes do, however, have control over their dietary intake, both habitually and surrounding exercise. Nutritional strategies involving fermentable carbohydrate restriction may be able to be applied to endurance athletes, as carbohydrates are frequently consumed during long-distance events to “improve endurance capacity and performance” and support glycogen synthesis and recovery (22, 23). If the types of carbohydrates consumed habitually and surrounding exercise are high in FODMAP, this could be contributing to athlete GI symptoms.

Recent research has indicated that athletes may already be altering their diets to eliminate some FODMAP-containing foods to reduce GI distress without intentionally restricting FODMAPs (24). Two case studies and three interventions have further suggested potential for a low-FODMAP diet in athlete GI symptom management (25–29) and low FODMAP diets are being implemented for up to 24 h prior to some interventions to minimize confounding effects on GI symptoms (30–34). Despite the promising preliminary work and the use of this diet as a lead in for performance research studies, the FODMAP content of foods consumed during exercise has received little attention. Athletes already desiring to consume low FODMAP products have little information on the FODMAP content of many popular sports nutrition products and examining ingredient statements may not be enough to predict FODMAP content (35). Furthermore, although Lis (36) reported high FODMAP foods commonly consumed by athletes, the list did not appear to be based on data collected from endurance athletes, either assessing habitual diets or nutritional habits prior to exercise.

To date, there has not been a larger scale assessment of endurance athlete diets or sports nutrition product usage from a FODMAP perspective, particularly in conjunction with GI symptoms. Many sports nutrition products have not yet been analyzed for FODMAP content, so athletes' actual FODMAP intakes may be difficult to determine. Additionally, while athletes may be already restricting some FODMAPs (24), their habitual intake levels and the association between intakes and GI symptoms are unknown. As such, the overall objective of this study was to evaluate the dietary habits of endurance athletes in relation to FODMAPs, both surrounding exercise and habitually. The primary aim was to examine athlete nutrition surrounding training and competition in order to determine the prevalence of high FODMAP food consumption. The secondary aim was to determine the habitual FODMAP intake of endurance athletes and explore relationships between this intake and GI symptoms. It was hypothesized that athletes commonly consume high FODMAP foods, both as pre-race meals and products consumed during exercise. It was further hypothesized that habitual FODMAP intakes would be high and associated with GI symptoms.

## Materials and Methods

Portions of the previously validated, online Endurance Athlete Questionnaire (EAQ) (37) were used in this study, as implemented in a recent assessment of IBS among endurance athletes (4). A total of 430 athletes who had completed or would complete a marathon, ultra-marathon, half-distance triathlon, or full-distance triathlon that year completed the EAQ. Participant recruitment and demographics are detailed in the previous work (4). The nutrition-related portions of the EAQ were utilized in this study and the symptom frequencies previously reported (4) were used to examine associations between nutrition and GI symptoms. The nutrition-related portions included general nutrition habits, foods consumed during pre-race dinners and breakfasts, and nutrition used 2 h prior to and during training and competition, specifically the frequency of use of water, sports drink/thirst quencher, sports drink/energy drink, solid food, gel/gummy, and homemade product/something else. When examining relationships with symptoms, athletes with previously diagnosed GI conditions which have overlapping symptoms with IBS (colitis, Crohn's, H. pylori/ulcers, celiac disease) were excluded from the analysis (*n* = 417), while those with diagnosed or undiagnosed IBS were included as defined in the previous work (4). This was done in order to examine dietary habits from a FODMAP perspective among athletes who it can already be assumed may benefit from FODMAP restriction (those with previously diagnosed IBS) and those whom this dietary strategy may have further application to (symptomatic athletes), as well as the endurance athlete population as a whole. This study was exempt from full Institutional Review Board (IRB) review after IRB review (protocol #16428; 7 December 2015) by the University of Illinois IRB and the Office of Protection of Research Subjects (Champaign, IL). Informed consent was obtained from all participants completing the EAQ through an online verification of consent (with a waiver of documentation of informed consent).

### Nutrition Surrounding Exercise

High FODMAP containing foods (for pre-race dinner and breakfast) were classified as specific high FODMAP if the athlete specifically named a high FODMAP food identified by the Monash University Low FODMAP Diet App (38). Pizza, pasta, bread, sweet potato, and avocado were examples of high FODMAP dinner foods while examples of foods at breakfast included bagel, toast, bread, orange juice, and yogurt. Entries into the questionnaire free response for dinner and breakfast were classified as potentially high FODMAP if they included either a specific high FODMAP food, a less specific food item that could potentially be high FODMAP, or an item that may be high FODMAP based on its ingredients but had not been analyzed yet. Examples include responses like “carbs,” “anything,” “veggies,” “fruit,” or sports nutrition products.

Athletes listed specific products that they used in the five categories of sports drink/thirst quencher, sports drink/energy drink, solid food, gel/gummy, and homemade product/something else. The first four categories of products were tabulated to determine the five most commonly used products in each category (six in the case of solid foods). The homemade product/something else foods were categorized as potentially high FODMAP in a similar way to the dinner and breakfast foods.

### Sports Nutrition Product Analysis

Product selection for FODMAP analysis was based on the three most frequently used product categories during competition, other than water. The top five sports drinks/thirst quenchers were selected. Although only one of these five was potentially high FODMAP based on review of the ingredients list, it was important to verify this assumption. From the solid food category, three of the top six products were selected due to their commercial availability. The other three products in this category were not specifically sports nutrition products (banana, peanut butter and jelly sandwich, pretzels), so data from these products can be found in the Monash University Low FODMAP Diet App or calculated by entering a recipe into the Monash University FODMAP Calculator to assess the FODMAP content, if desired (26, 39). An additional solid product was added for FODMAP analysis due to it being a gluten-free version of the second most popular solid product. The top six products from the gel/gummy category were selected; however, one brand was discontinued prior to FODMAP analysis, so four gel brands and one gummy product were analyzed. Two flavors of two gel brands were tested to show potential variation in FODMAP content based on flavor and ingredient differences. Product ingredient lists are shown in Supplementary Table 1.

Validated, standardized, and published methods for FODMAP analysis were conducted by researchers at Monash University (40–42). Samples of products were obtained based on Monash testing methods as described previously, which included samples from three different product batches (40, 42, 43). Processing and extraction methods were followed (40, 42, 43). In summary, ~100–200 g from each of three sample batches was randomly selected, pooled, and mixed. Three samples were freeze dried after which samples were homogenized by grinding into powder. Non-freeze dried samples were ground or mixed into a uniform mixture. From each of these powdered or mixture forms, 1 g was extracted in triplicate. Analysis of fructose, mannitol, sorbitol, lactose, and galacto-oligosaccharides (GOS) involved high performance liquid chromatography (HPLC) with evaporative light scattering detection (ELSD) as described in previous FODMAP analysis research (40, 41, 43). Total fructans were analyzed by enzymatic kit (Megazyme Fructan HK Assay kit; Megazyme International Ireland Ltd, Wicklow, Ireland; AOAC Method 999.03 and AACC Method 32.32). Averages of the triplicate measurements were calculated, with data reported on a per serving basis based on the serving size of each product. Low FODMAP cutoff values are based on the amount of each FODMAP type in a product consumed in one sitting (35). A sitting or meal is typically separated by 2–3 h; however, athletes commonly consume multiple servings of products in shorter time spans since 30–90 g/h are recommended for endurance and ultra-endurance exercise (23). Thus, the number of servings consumed in one sitting that would result in the product exceeding the low FODMAP cutoff values was also calculated (35).

### Habitual Nutrition

Athletes who had previously completed the EAQ and had given permission to be contacted regarding additional studies were eligible for participation in a follow up study using a FODMAP-specific food frequency questionnaire. The Comprehensive Nutrition Assessment Questionnaire (CNAQ) has previously been shown to be valid assessment of dietary FODMAP (39). The CNAQ was administered through an online portal controlled by the researchers which sent instructions and follow-up emails to participants (http://www.cnaq.com.au). Qualifying athletes (*n* = 234) were contacted with details of the follow up study, 120 consented to participate, and 80 completed the CNAQ. Three participants were excluded from the final analysis for reported daily intakes of >5,000 kcal (44). Four were excluded due to previously diagnosed GI conditions as in the EAQ analysis (two with inflammatory bowel disease, one with celiac disease, and one with H. pylori/ulcers), for a final sample size of 73 athletes, with demographic information shown in Table 1. Classification of IBS was based on medical diagnosis (*n* = 5) plus athletes meeting the Rome III criteria for IBS (*n* = 8), the most commonly used diagnostic criteria at the time (4). This portion of the study was exempt from full Institutional Review Board (IRB) review after IRB review (protocol #17260; 21 November 2016) by the University of Illinois IRB and the Office of Protection of Research Subjects (Champaign, IL). Corresponding data from the EAQ on GI symptoms, including frequency of GI cramps/pain, bloating, flatulence, urge to defecate, defecation, diarrhea, and constipation, were utilized for each athlete by using random 5-digit identifiers.

**Table 1:** Comprehensive nutrition assessment questionnaire (CNAQ) endurance athlete participant demographics (*n* = 73).

**Characteristic**	***n***	**%**
**Sex**		
Male	31	42.5
Female	42	57.5
**Age**		
18–29	17	23.3
30–39	23	31.5
40–49	18	24.7
50–59	12	16.4
60+	3	4.1
**Ethnicity**		
American Indian or Alaskan Native	1	1.4
Black/African American	1	1.4
Caucasian	66	90.4
Hispanic/Latino	1	1.4
>1 Ethnicity	4	5.5
**Competition level**		
Beginner/amateur/casual	36	49.3
Competitive age-grouper	35	47.9
Elite/professional	2	2.7
Lifetime competition participation*		
Marathon	59	80.8
Ultra-marathon	23	31.5
Half-distance triathlon	40	54.8
Full-distance triathlon	25	34.2
**BMI category**		
Underweight (<18.5)	2	2.7
Normal (18.5–24.9)	57	78.1
Overweight (25–29.9)	14	19.2
**IBS classification**		
Non-IBS	60	82.2
IBS	13	17.8

* *Athlete participants were able to select all event types previously completed*.

*Defined as medically diagnosed or fitting Rome III diagnostic criteria*.

Average intake values for all FODMAP categories were calculated. Total daily FODMAP intake (both including and excluding lactose) was categorized as low (≤12 g) or high FODMAP based on levels shown to have therapeutic benefit in clinical trials (45). Using the methods of O'Keeffe et al. (46), the individual foods on the CNAQ were categorized as high and low in each FODMAP (fructans, GOS, lactose, fructose, sorbitol, and mannitol). The proportion of athletes with a regular intake (at least once a week) of at least one food high in individual FODMAP was assessed (46).

### Statistical Analysis

A Shapiro-Wilk test was performed to assess normality of product consumption frequencies, symptom frequencies, and FODMAP intakes. Non-parametric statistical analysis was performed where appropriate due to non-normal distributions. The relationship between nutrition product type consumption frequency and symptom frequency at various time points was not monotonic, so Spearman's correlation between the two was not valid. Instead, a Kruskal-Wallis test was conducted to determine if there were differences in symptom frequency between groups that differed in their nutrition product type intake frequency. Distributions of symptom frequencies were not similar for all groups, as assessed by visual inspection of boxplots. A total of 252 associations between nutrition product type frequency and GI symptom frequency at each time point were tested. When significant differences in the omnibus Kruskal-Wallis H test statistic were found, Dunn's procedure for *post-hoc* pairwise comparison with Bonferroni correction was used to determine differences in symptom frequency between intake levels.

Individual symptom frequencies were dichotomized with never and rarely as not present and sometimes, often, and always as present (4). If at least one symptom was present during a certain timepoint (rest, during training, during competition, 2-h after training, and 2-h after competition), the participant was classified as having symptom(s) at that time point. Mann-Whitney U tests were used as a non-parametric independent-samples *t*-test alternative comparing dichotomized symptom presence and continuous FODMAP intakes as well as IBS classification and continuous FODMAP intakes. Fisher's Exact test or Pearson Chi-Squared test were conducted between total FODMAP classifications (high or low) and dichotomized symptom presence. Additionally, Mann-Whitney U tests were conducted to compare the individual symptom frequencies between those reporting low or high habitual FODMAP intakes.

All analyses were performed using SPSS software package version 25 (IBM SPSS Software, NY, USA) with results of all tests considered significant if *p* < 0.05.

## Results

**Figure 1:**
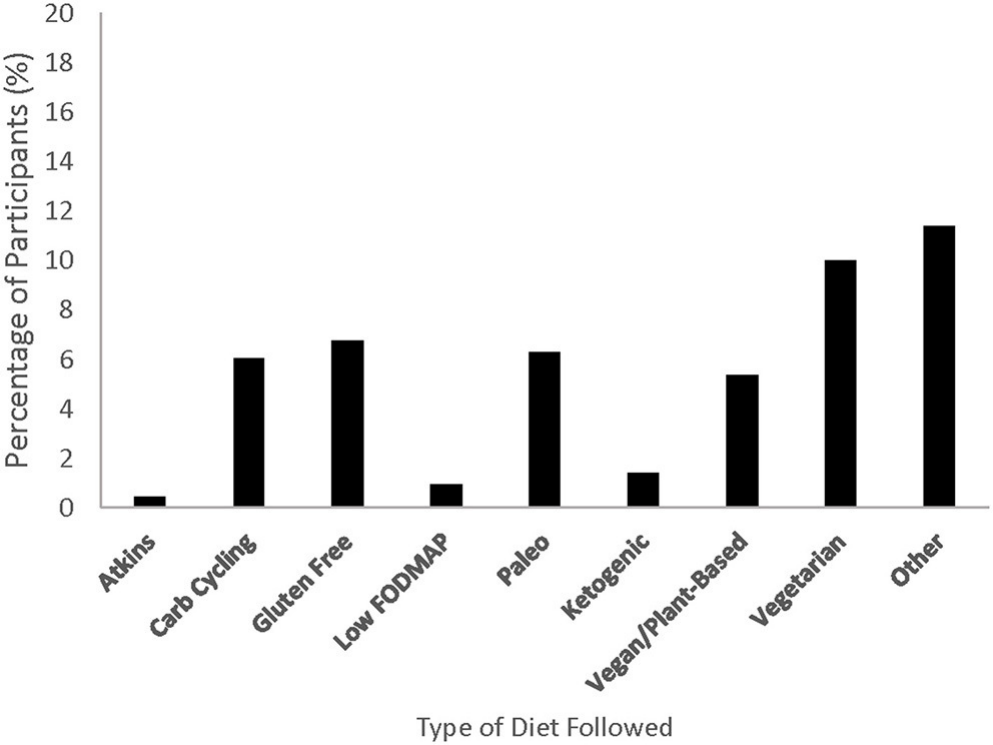
Percentage of endurance athletes (*n* = 430) following various specific diets. FODMAP, fermentable oligosaccharides, disaccharides, monosaccharides, and polyols.

The percentage of athletes who reported following various diets is shown in Figure 1, with a total of 34.7% of athletes reporting at least one type of specific diet. Additionally, 65.3% reported following no specific diet. When asked how important their nutritional strategy is for training and competition, 23.3 and 66.0% of athletes reported that it is somewhat or very important, respectively. Athletes sometimes (21.9%), often (30.2%) and always (36.5%) used the race-provided foods/beverages during a race either from race packets or aid stations. The percentage of athletes reporting either a specific or potentially high FODMAP food for pre-race dinner was 65.5 and 87.2%, respectively. In terms of pre-race breakfast foods, 62.3 and 85.1% reported a specific or potentially high FODMAP food, respectively.

**Figure 2:**
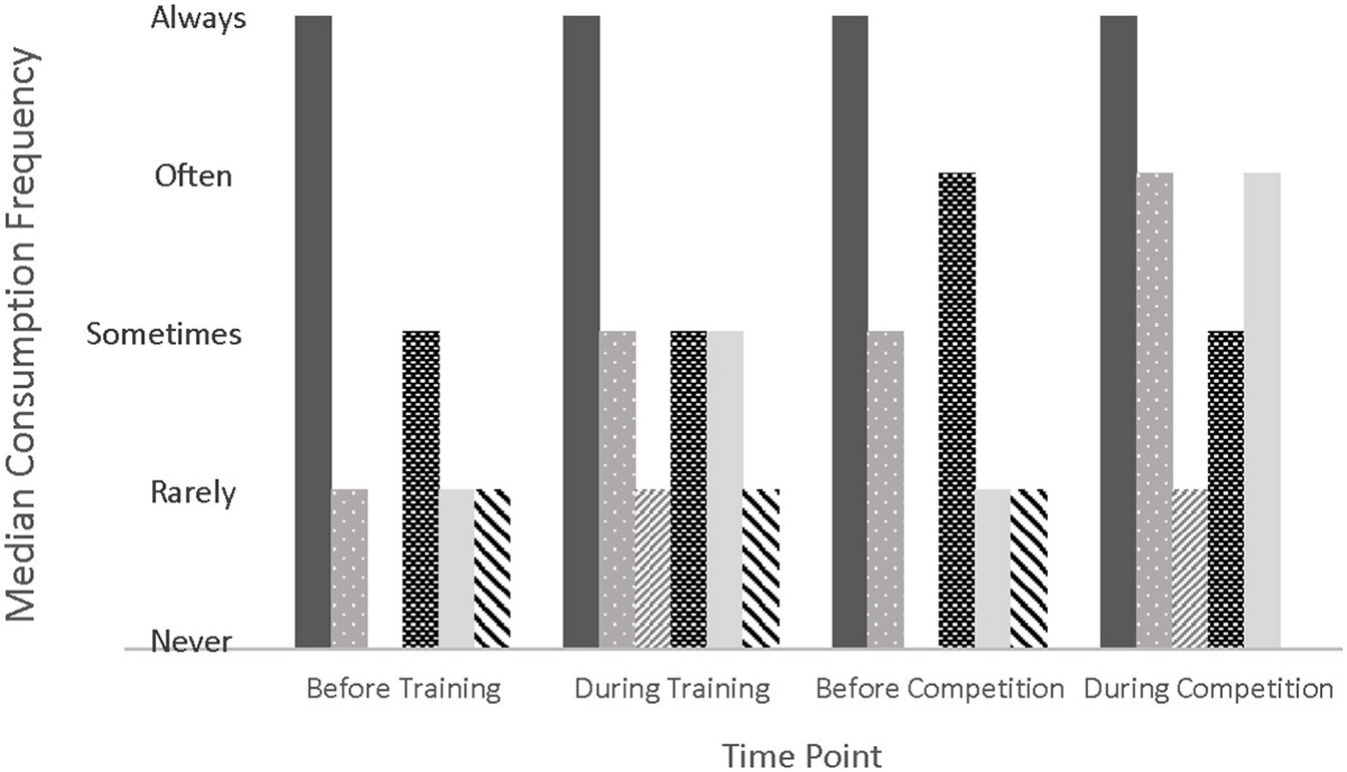
Median consumption frequency of nutrition product categories at various time points surrounding exercise. Product categories include water (solid dark gray), sports drink/thirst quencher (light gray with polka dot), sports drink/energy drink (light gray diagonal stripe), solid foods (black with white rectangles), gel/gummy (solid light gray), homemade product (black diagonal stripe).

Figure 2 displays the consumption frequency of water, sports drink/thirst quencher, sports drink/energy drink, solid food, gel/gummy, and homemade product/something else before and during training and competition. When these frequencies were analyzed in conjunction with the symptom frequencies, there were some significant differences at different levels of intake frequency (Supplementary Tables 2–9). *Post hoc* analysis revealed 69 significantly different pairs, 55 of which had a higher symptom frequency (reported as mean rank) with higher intake frequency. Associations with the more problematic symptoms (GI cramps/pains, urge to defecate, defecation, and diarrhea) are shown in Table 2.

**Table 2:** Associations of nutrition product category consumption frequency with more problematic gastrointestinal (GI) symptom frequency surrounding training and competition.

**Product category**	**Symptom**	**Consumption timing**	**Symptom timing**	Significant relationships*
Sports drink/thirst quencher	GI cramps/pain	Before training	During training	Always < All others
	Urge to defecate	Before training	During training	Always < Never, Rarely, & Sometimes
Sports drink/Energy drink	Diarrhea	Before training	2 h after training	Rarely > Never
Solid food	GI cramps/pain	Before training	During training	Often > Never
	GI cramps/pain	During training	During training	Always < All others†
	Urge to defecate	Before competition	2 h after competition	Sometimes > Never
	Defecation	Before competition	2 h after competition	All others > Never
Gel/gummy	GI cramps/pain	Before training	During training	Always < Sometimes†
	Urge to defecate	During training	During training	Sometimes > Never
	Urge to defecate	During training	2 h after training	Sometimes, Often, & Always > Never
	Defecation	During training	2 h after training	Sometimes, Often, & Always > Never
	Urge to defecate	During competition	During competition	Sometimes & Often > Never
Homemade product/something else	GI cramps/pains	Before training	2 h after training	Always > Never
	GI cramps/pains	During training	2 h after training	Rarely > Never
	GI cramps/pains	Before competition	During competition	Sometimes > Never
	Defecation	Before competition	During competition	Sometimes > Never
	GI cramps/pain	Before competition	2 h after competition	Sometimes > Never
	GI cramps/pain	During competition	2 h after competition	Rarely > Never

* *Relationships shown are product consumption frequencies based on Kruskal-Wallis H test with post hoc pairwise comparisons using Dunn's procedure and Bonferroni correction comparing mean ranks. A < B means the mean rank of A was less than the mean rank of B. A > B means the mean rank of A was greater than mean rank of B. Details of relationships are shown in Supplementary Tables 2–9*.

*Highlights relationships in which more frequent intake was associated with less frequent symptom*.

The tested products in the categories of sports drink/thirst quencher, solid, and gel/gummy were classified as low or high FODMAP based on the FODMAP analysis results and established cutoffs (35), with details shown in Table 3. The use frequency of the top reported products in these categories during competition is also displayed in Table 3 as the popularity. Other products among the top five in the solid category included bananas (12.6%) and peanut butter and jelly sandwiches (7.0%). Products in the sports drink/energy drink category were consumed less often, with the top five reported products only used by up to 5.3% (23 of 430) of athletes. Of the 140 athletes who reported using a homemade product/something else during training, 64.3% reported a potentially high FODMAP product. Additionally, of the 122 athletes who reported using a similar product during competition, 63.9% were potentially high FODMAP. Commonly reported potentially high FODMAP homemade foods included sandwiches and bites or bars made with dried fruit and/or honey.

**Table 3:** Specific fermentable oligosaccharides, disaccharides, monosaccharides, and polyols (FODMAP) composition of commonly used beverage, solid, and gel/gummy products.

**Product**	**Total fructans**	Total GOS* (raffinose + stachyose)	**Lactose**	**Fructose**	**Glucose**	**Excess fructose**	**Sorbitol**	**Mannitol**	**Total Polyol**	**Total FODMAP**	**FODMAP rating**	**# of servings for high FODMAP**	**Popularity (%)******
Bev1 g per serving (355 mL)	0.39	0.00	0.00	4.37	4.89	0.00	0.00	0.00	0.00	0.39	High	1	45.6
Bev2 g per serving (360 mL)	0.03	0.00	0.00	5.71	3.58	2.14	0.00	0.00	0.00	2.16	High	1	7.7
Bev3 g per serving (5.2 g)	0.00	0.00	0.00	0.00	0.35	0.00	0.00	0.00	0.00	0.00	Low	–	7.4
Bev4 g per serving (27 g)	0.07	0.00	0.00	0.00	10.66	0.00	0.00	0.00	0.00	0.07	Low	3	7.2
Bev5 g per serving (22 g)	0.04	0.00	0.00	0.06	1.08	0.00	0.00	0.00	0.00	0.02	Low	5	7.0
Bar1 g per serving (68 g)	0.16	0.06	0.00	0.63	4.77	0.00	0.01	0.00	0.01	0.23	High	1	14.0
Waf1 g per serving (30 g)	0.13	0.00	0.00	0.36	1.63	0.00	0.00	0.00	0.00	0.13	Low	2	8.8
Bar2 g per serving (49 g)	0.40	0.11	0.00	0.84	3.99	0.00	0.12	0.00	0.12	0.62	High	1	4.0
Waf2 g per serving (30 g)	0.06	0.00	0.00	0.06	1.15	0.00	0.00	0.00	0.00	0.06	Low	4	N/A
Gel1 g per serving (32 g)	0.03	0.12	0.00	4.82	0.12	4.71	0.00	0.00	0.00	4.85	High	1	38.1
Gel2A g per serving (34 g)	0.14	0.00	0.00	6.67	5.64	1.04	0.00	0.00	0.00	1.18	High	1	11.9
Gel2B g per serving (32 g)	0.05	0.00	0.00	0.26	3.08	0.00	0.00	0.00	0.00	0.05	Low	4	11.9
Gel3A g per serving (33 g)	0.04	0.11	0.00	0.07	0.24	0.00	0.00	0.00	0.00	0.16	Low	2	11.2
Gel3B g per serving (33 g)	0.03	0.00	0.00	0.28	0.25	0.03	0.01	0.00	0.01	0.06	Low	5	11.2
Gel4 g per serving (34 g)	0.03	0.00	0.00	0.03	0.12	0.00	0.00	0.00	0.00	0.03	Low	7	10.9
Gum1 g per serving (30 g)	0.10	0.13	0.00	0.19	0.56	0.00	0.00	0.00	0.00	0.23	High	1	16.0

* *GOS, galacto-oligosaccharides*.

*Indicates serving size of a powder or tablet that is intended for dissolution in 350–500 ml water*.

*Popularity as percentage of athletes (n = 430) reporting use of brand during competition. Not necessarily same flavor as tested*.

*Sports drink/thirst quenchers are abbreviated with Bev (for beverage), solids with either Bar or Waf (for waffle), and gel/gummies with either Gel or Gum (for gummy)*.

Table 4 shows the individual habitual FODMAP intakes of endurance athletes, with general nutritional information shown in Supplementary Table 10. The percentages of athletes who consumed at least one high FODMAP food in each category at a frequency of at least once a week are shown in Table 5. There were no significant differences in individual FODMAP intakes between athletes with or without IBS. Additionally, there were no significant differences in FODMAP intakes between those experiencing or not experiencing at least one symptom at each time point. When examined at the symptom level, there were significant differences in some FODMAP intakes among athletes with or without specific symptoms. Distributions of FODMAP intakes for those with and without symptoms were similar, as assessed by visual inspection. Significantly different median FODMAP intakes are shown in Table 6, together with the associated symptoms.

**Table 4:** Habitual fermentable oligosaccharides, disaccharides, monosaccharides, and polyols (FODMAP) intakes of endurance athletes (*n* = 73).

	**Daily Intake (g)**			
**Specific FODMAP**	**Mean ± SD**	**Min**	**Max**	Median (IQR)*
Total Oligosaccharides	4.4 ± 2.3	1.0	11.5	3.8 (3.0)
Fructooligosaccharides	3.0 ± 1.5	0.6	6.7	2.8 (1.8)
Galacto-oligosaccharides	1.4 ± 1.1	0.1	5.8	1.1 (1.2)
Raffinose	0.6 ± 0.5	0.0	2.4	0.5 (0.7)
Stachyose	1.2 ± 1.0	0.0	5.3	0.9 (0.8)
Lactose	13.5 ± 13.8	0.0	80.1	11.4 (13.1)
Excess Fructose	5.0 ± 6.6	0.2	34.0	2.9 (3.9)
Total Polyols	3.3 ± 2.4	0.5	14.9	2.5 (3.3)
Mannitol	0.9 ± 0.8	0.1	5.4	0.8 (0.8)
Sorbitol	2.4 ± 2.0	0.2	9.8	1.9 (2.5)
Total FODMAP	26.1 ± 15.9	3.0	100.9	23.0 (18.9)
Total FODMAP (not lactose)	12.7 ± 9.2	2.9	54.6	11.5 (8.5)

* *Median interquartile range (IQR) listed for comparison to Mann-Whitney U statistic results of intake differences between athletes with and without certain symptoms in Table 6*.

**Table 5:** Percentage of endurance athletes (*n* = 73) consuming high fermentable oligosaccharides, disaccharides, monosaccharides, and polyols (FODMAP) foods at least once a week.

**Specific FODMAP**	**Consumed at least once a week (% of athletes)**
Fructans	98.6
Galacto-oligosaccharides	86.3
Lactose	83.6
Excess Fructose	97.3
Mannitol	89.0
Sorbitol	87.7

**Table 6:** Significant differences in median fermentable oligosaccharides, disaccharides, monosaccharides, and polyols (FODMAP) intakes between individual symptom presence categories^*^.

**Symptom**	**FODMAP**	**Symptom not present, median (*n*)**	**Symptom present, median (*n*)**	***U***	***z*******	***p*^§^**
Constipation during rest	Stachyose	0.8 (63)	1.3 (9)	434.0	2.569	0.010
	Mannitol	0.8 (63)	1.3 (9)	402.5	2.030	0.042
Defecation during training	Total Polyols	2.4 (52)	4.9 (20)	693.5	2.182	0.029
	Mannitol	0.8 (52)	1.1 (20)	676.0	1.965	0.049
Diarrhea during training	Stachyose	0.8 (57)	1.2 (15)	599.0	2.384	0.017
Constipation during training	Total Oligosaccharides	3.5 (65)	7.7 (6)	324.5	2.678	0.007
	Fructooligosaccharides	2.7 (65)	5.3 (6)	336.5	2.927	0.002
	Galacto-oligosaccharides	1.0 (65)	2.8 (6)	316.0	2.505	0.010
	Raffinose	0.4 (65)	1.1 (6)	305.5	2.296	0.019
	Stachyose	0.9 (65)	2.3 (6)	340.5	3.016	0.001
	Total Polyols	2.4 (65)	5.7 (6)	323.5	2.657	0.006
	Mannitol	0.7 (65)	1.5 (6)	314.5	2.476	0.011
	Sorbitol	1.7 (65)	1.5 (6)	308.5	2.348	0.016
Constipation during competition	Total Oligosaccharides	3.5 (65)	8.3 (5)	265.5	2.350	0.015
	Fructooligosaccharides	2.7 (65)	5.3 (5)	273.0	2.521	0.009
	Galacto-oligosaccharides	1.0 (65)	3.0 (5)	264.5	2.330	0.017
	Stachyose	0.9 (65)	2.3 (5)	295.0	3.030	0.001
	Total Polyols	2.4 (65)	5.7 (5)	279.5	2.669	0.005
	Mannitol	0.7 (65)	1.6 (5)	261.0	2.251	0.022
	Sorbitol	1.8 (65)	3.9 (5)	266.0	2.362	0.015

* *Sample size for each symptom tested varied due to limited missing data and can be calculated by the sum of n for symptom not present and symptom present*.

*Mann-Whitney U statistic*.

*z-score from Mann-Whitney U test*.

§ *p-value from Mann-Whitney U test; significant at p < 0.05*.

When using an overall FODMAP cutoff value of 12 g including lactose, 11 of 73 athletes (15.1%) had habitual diets low in FODMAP while 62 (84.9%) consumed diets high in FODMAP. When excluding lactose in this 12 g cutoff, 40 athletes (54.8%) and 33 athletes (45.2%) consumed low and high FODMAP daily diets, respectively. There were no significant associations between FODMAP categorization including lactose and overall symptom presence at any time point or individual symptom presence. Similarly, there were no significant associations between FODMAP categorization excluding lactose and overall symptom presence at any time point. There was a significant moderate association between FODMAP categorization excluding lactose and the presence of defecation presence during training [χ^2^ = 4.098,(1) *p* = 0.043, ϕ = 0.239] (47). Of those consuming high FODMAP diets, 39.4% (13 of 33) experienced defecation during training while only 17.9% (7 of 39) of those in the low FODMAP group experienced this symptom. Differences in non-dichotomized individual symptom frequencies were examined between FODMAP categorizations with similar distributions between low and high categories, as assessed by visual inspection. Both post-training (*U* = 871, *z* = 2.833, *p* = 0.005) and post-competition (*U* = 850, *z* = 2.602, *p* = 0.009) bloating median frequencies were significantly greater for the high FODMAP group (median = 1.0; rarely) compared to the low FODMAP group (median = 0.0; never) when considering total FODMAP excluding lactose.

## Discussion

Overall, this study showed that FODMAP intake is high among endurance athletes both surrounding exercise and habitually. Only four athletes of 430 reported following a low FODMAP diet, despite twelve athletes having been diagnosed with IBS by a medical professional (4). Athletes commonly reported consuming high FODMAP foods as part of pre-race dinners and breakfasts. Many of the most commonly reported sports nutrition products contained high levels of FODMAP, particularly as servings increase beyond one portion. Furthermore, out of 73 athletes, 62 (84.9%) had a total daily FODMAP intake (including lactose) at a level that would be considered high FODMAP (45). When lactose intake was excluded to calculate athletes at or below the 12 g cutoff, there were still over 45% of athletes who consumed daily diets high in FODMAP (45). There were various significant relationships between sports nutrition product type usage frequency and symptom frequency, many of which displayed increased symptom frequency with additional product frequency. This was also true for relationships between FODMAP intake and symptoms, with more symptoms present in those with higher FODMAP intakes. Higher median intakes were also shown among those with individual symptoms present.

The diets reported by athletes in this study may have varying effects on their habitual FODMAP intake and may also impact food choices surrounding exercise. Athletes following a vegetarian (10.0%) or vegan/plant based (5.3%) diet commonly consume vegetables, fruits, grains, legumes, and nuts, which have the potential to contain significant levels of FODMAPs (48). Alternatively, following a gluten free (6.7%) or ketogenic (1.4%) diet may necessitate selecting daily foods, pre-race meals, or products for use during exercise that are limited in at least some FODMAP. Athletes following a paleo diet (6.3%) fall somewhere in between these two groups, as potentially high FODMAP fruits, vegetables, and nuts are consumed but lower FODMAP dairy, legumes, and grains are not.

### Nutrition Surrounding Exercise

High FODMAP pre-race dinners were common, with many of the reported dinners containing a wheat-based product (bread, pasta, pizza) likely with high levels of fructans (40). A dietary survey of ultraendurance cyclists similarly reported 89% of participants consuming pasta, pizza, or both within the 3 days leading up to a 210 km cycle race (49). Additionally, many of these products include sauces which typically contain onion and garlic, which are also high in fructans (42). The same was true for many of the high FODMAP breakfast foods, including wheat-based bagels and toast. Bananas were also a common breakfast food, which may be high in fructans depending on the size and ripeness. These foods were also the top four sources of fructans (wheat, onion, banana, garlic) in a study of fructan intake of patients with Crohn's disease (50). Similar high FODMAP foods and ingredients were listed as commonly consumed in recent reviews of low FODMAP diets and athletes (36, 51) and pre-race dinners and breakfasts are similar to those reported by ultraendurance cyclists (49).

Water was the most commonly consumed product at all time points and showed no relationships with GI symptoms. Lower hydration levels and dehydration have been related to increased GI symptoms in ultramarathon and marathon runners (52, 53). In this study, reported intake levels were likely high enough in the majority of athletes that relationships were not seen. Also, athletes who consume water less frequently may use alternate sources of fluids such as a sports drink/thirst quencher. These products were used frequently by athletes, particular during exercise, and were associated with less GI cramps/pain and urge to defecate during training when consumed before training. Three of the top five products in this category were low FODMAP, so athletes may be able to consume additional fluid, typically with carbohydrates and electrolytes, without consuming additional FODMAP. Contrastingly, in a similar study of endurance athlete nutritional habits by Peters et al. (5), thirst quenchers were found to be related to an increased relative risk of GI symptoms. Other studies have also shown increased GI symptoms due to carbohydrate sports drink consumption (54, 55), with factors such as osmolality and impaired intestinal absorption due to running as hypothesized causes. It is important to note that the two most commonly used products in this category tested as high FODMAP in one serving. While this present study did not find the same relationships, this new FODMAP information may help to explain the previous findings (5).

Frequency of consuming sports drinks/energy drinks was fairly low in this population despite other reports of common use as in endurance sports and benefits to endurance performance (56–58). Intake of these beverages prior to training was associated with diarrhea after training and some other minor GI symptoms. This is contrary to previous work that demonstrated no effect on GI symptoms with addition of caffeine to a sports drink in athletes completing an 18-km run (55). Despite this observation, caffeine is included in lists of supplements to be avoided before and during exercise for athletes who experience GI symptoms such as diarrhea (59, 60). Due to the potential laxative effect of caffeine, restriction of caffeinated beverages is commonly recommended in dietary guidelines for IBS, while their inclusion is recommended to promote motility in patients with constipation (12, 61, 62).

Both solids and gels were related to various GI symptoms, including half of the more problematic symptoms. These products were more commonly used than sports drink/energy drinks or homemade products. Previous research on nutritional habits of ultraendurance cyclists showed common consumption of these two forms of carbohydrate, along with carbohydrate beverages, particularly during races (49). In a study of nutrition during various endurance events, gels were found to account for 28–45% of carbohydrate intake during an amateur long-distance cycling race and marathon, respectively (63). Solids accounted for 15% of carbohydrate intake during and Ironman triathlon race and 37% of intake by professional cyclists (63).

Gels in particular are a common and convenient way for athletes to ingest high levels of carbohydrate (49, 64, 65). Previous research on gel use and GI discomfort has shown mixed results. In general, gels are well tolerated by most athletes (64, 66); however, a few athletes experience significant GI symptoms (64, 67). In this study, more frequent gel/gummy use during both training and competition was correlated with more frequent urge to defecate in some groups compared to those who never used these products. Of the six gels and one gummy tested for FODMAP content, three were high FODMAP in one serving and the others became high after two, four, five, and seven servings, so it is possible that the FODMAPs in these products are contributing to symptoms, particularly the oligosaccharides and excess fructose.

More frequent solid product use before training was associated with more frequent abdominal cramps/pain during training in this study. Peters et al. (5) also found solid product use to be associated with lower GI symptoms and a study of different carbohydrate formats during intense cycling showed that abdominal cramps were more likely when bars were consumed compared to gels (66). Of the solid products tested for FODMAP content, two were high FODMAP in one serving and the other two were high after two and four servings. The other top products listed by athletes in this category were banana, peanut butter and jelly sandwich, and pretzels, all of which are potentially high FODMAP depending on factors such as ingredients, processing, and serving sizes (35, 40, 43, 48, 50).

Alternatively, athletes who reported always consuming solid food during training experienced less frequent GI cramps/pain during training compared to all other groups. The same was true for those always consuming gel/gummies before training compared to sometimes. In these cases, it is possible that athletes who always consume these products experienced training of the gut to be able to tolerate higher levels of carbohydrates without adverse GI symptoms, although previous work in this area was not specific to FODMAP (34, 60, 68).

Many of the products in the sports drink/thirst quencher, solid, and gel/gummy categories are made available to athletes at races either in race packets or at aid stations (24). The majority of athletes reported using the products available, although it is unclear how often they train with the same products. This could be problematic in race situations as athletes may be consuming products high in FODMAP when they are not used to training with them (6, 69).

Although less frequently used compared to other product categories, homemade products/something else were associated with many problematic symptoms, particularly GI cramps/pain, but also defecation during competition. This would very likely impact an athlete's performance as they would need to take the time to stop and use a restroom (70). Many of the products reported in this category were potentially high FODMAP and although there was a wide range of different products reported, many shared common high FODMAP ingredients (24, 51). Low FODMAP ingredients such as maple syrup and coconut sugar may be able to be used in place of honey. Athletes may also want to consider limiting amounts of dried fruits which typically become high FODMAP at larger serving sizes.

### Habitual Nutrition

FODMAPs may contribute to GI symptoms experienced by an individual when the level of specific FODMAPs consumed surpass that individual's unique thresholds. If an athlete habitually consumes a diet low in FODMAP, then consumption of high FODMAP products surrounding training and competition may not be in sufficient quantities to elicit symptoms, since they started out well below their threshold. Alternatively, if their habitual diet is already high in FODMAP, they may already have high enough levels to elicit symptoms or the additional FODMAP from sports nutrition products could tip the scales.

This research demonstrated that, in general, endurance athletes' habitual diets are high in FODMAP and consumption of foods high in each FODMAP type is common. Limited application of the relatively new CNAQ and no previous research detailing the individual FODMAP intakes of more than one athlete make comparisons difficult for this present work. FODMAP intake appears to be consistent with the high levels seen in habitual diets of the general population and other studies of individual athletes (14, 18, 26, 27, 46). The total daily FODMAP level seen here (26.1 g) was similar to that in the typical Australian diet (23.7 g) used as a high FODMAP interventional diet in a study of the effects of a low FODMAP diet on IBS symptoms (14). It was also similar to the habitual diet FODMAP intakes (29.4 and 29.6 g) in studies on long-term low FODMAP diet effectiveness (46) and effects on microbiota in patients with IBS (18), respectively. Additionally, it is above the high FODMAP diet intake cut off (20 g) used to qualify participants in a study of low FODMAP diet effectiveness for athletes (27). In a recent study of the effects of a 7-day low FODMAP diet on exercise-related GI symptoms in recreational runners, average habitual FODMAP intake was 28.04 g, although it is unclear how the habitual diet was analyzed for FODMAPs in this case (28). Furthermore, the percentage of athletes consuming products of each FODMAP type at least once a week was generally 3-fold higher than those who went back to consuming a “habitual” diet in the study of long-term low FODMAP diet effectiveness, which also used the CNAQ to measure FODMAP intake (46). This indicates that while athletes are not necessarily consuming diets higher in overall FODMAP content than the general population, more athletes appear to commonly consume foods that are high in individual FODMAPs.

The lactose intake of endurance athletes was higher than all other FODMAPs in this study. In fact, lactose accounted for approximately half of the total FODMAP intake, which was also seen in other studies of habitual intakes (14, 46). There was also a wide range of lactose intakes among athletes. In a case study of a symptomatic endurance athlete who was placed on a low FODMAP diet, Lis et al. (26) discussed why dairy consumption may be higher among athletes; however, lactose-containing foods were also the most commonly restricted by athletes to help improve GI symptoms (24). Lactose intakes were not significantly different between those with or without symptoms at any time point. It is possible that because lactose intolerance is a more well-known issue, athletes who have GI trouble due to lactose are already restricting this FODMAP and those that do not have a problem are able to consume higher quantities (24). None of the sports nutrition products tested contained lactose.

Over two-thirds of oligosaccharide consumption was as fructooligosaccharides or fructans, which is consistent with common consumption of the high fructan-containing foods during pre-race dinners and breakfasts reported on the EAQ. Additionally, 15 of the 16 products tested contained some level of fructans and five products contained GOS. Importantly, the most common sports nutrition beverage, both bars, and the gummy all tested high in oligosaccharides. The source of these FODMAPs is clear in some cases (soy or wheat ingredients), but less so in others (potentially gums or maltodextrins) (38). Comparison of dichotomized symptom presence showed that total oligosaccharide, fructan, and GOS intakes were higher in athletes experiencing constipation during training or competition, with raffinose intakes greater during the former and stachyose during the latter. Stachyose intakes were also higher among athletes experiencing constipation at rest, and diarrhea during training. This indicates that high habitual intakes, coupled with high levels of these FODMAPs in popular sports nutrition products, could be contributing to symptoms.

Foods high in excess fructose were also commonly consumed by athletes. There were no significant differences in excess fructose intakes between athletes experiencing or not experiencing symptoms at various time points. This may be due to a similar reason as lactose, with athletes with known fructose malabsorption issues already restricting intakes. High fructose foods and beverages are typically not recommended for athletes since they have been shown to lead to GI distress (3, 71) and athletes have reported avoiding such foods (24). The top two most commonly reported gels (Gel1 and Gel2A) and the second most reported sports drink/thirst quencher (Bev2) were high in excess fructose. These products were not included in the CNAQ assessment, and may help to explain some of the relationships seen between increased frequency of more severe symptoms and increased consumption frequency of these product categories. The excess fructose in these products is likely coming from ingredients such as fructose, honey, apple juice (in a Gel 3B which contained excess fructose but was not high in one serving), and high fructose corn syrup (38).

Polyol intake had the lowest habitual consumption of the FODMAP categories, but higher intakes were found in those experiencing constipation at rest (mannitol), defecation during training (mannitol and total polyols), and constipation during training and competition (mannitol, sorbitol, and total polyols). Mannitol is a sugar alcohol that commonly occurs in fruits and vegetables such as button mushrooms, cauliflower, snow peas, sweet potatoes, and watermelon (43). This highlights a complex issue for athletes in relation to FODMAPs in their habitual diets, since FODMAPs such as mannitol are found in many whole foods. Individuals who exercise frequently also tend to engage in other health-related behaviors including eating a healthful diet (72, 73) which typically includes foods such as fruits, vegetables, whole grains, and low fat dairy. Furthermore, over 15% of endurance athletes reported following either vegan or vegetarian diets on the EAQ. These diets have the potential to be high in FODMAP, but by selecting specific foods and using certain processing techniques (cooking and straining, pickling, etc.), a vegetarian or vegan diet can still be low FODMAP (48). Only three of the tested sports nutrition products contained mannitol (two at very low levels; 0.01 g/serving) and none contained sorbitol, so it appears that consumption of these potentially problematic FODMAPs can easily be avoided during exercise.

When lactose was excluded from the overall FODMAP intake cut off of ≤12 g, some significant differences were seen in symptomology between low and high FODMAP habitual diets, including defecation during training and bloating after training and competition. This observation is similar to previous studies which found symptom improvement with FODMAP intake of ≤12 g and support this cut-off level of total FODMAP (14, 15, 45, 74, 75). The cut-off was analyzed by both including and excluding lactose due to habitually high lactose levels in this population and the likelihood that athletes sensitive to lactose are already excluding this FODMAP, as explained above and evidenced by the variable intakes found in this study (24, 26, 27).

### Limitations

While this study demonstrated the potential for FODMAPs to be playing a role in the GI symptoms of endurance athletes due to high usage rates and some relationships with symptoms, there are a few limitations. When a low FODMAP diet is implemented in a patient with GI symptoms such as with IBS, it is not intended as a long-term diet. Initially, all FODMAP are restricted and then categories of FODMAPs are reintroduced to find individual sensitivities and thresholds unique to each person (17, 76). That person can then safely consume the FODMAPs that do not cause symptoms while continuing to limit the ones that do. This highly individualized nature of FODMAPs makes drawing generalized relationships between foods or intakes and symptoms across multiple athletes difficult. Individuals likely do not have the same thresholds for the different FODMAP types, so some athletes may be able to consume high levels of specific FODMAPs while it may be problematic for other athletes. It is also currently unknown if or how the stressors of exercise affect the proposed cutoff level of 12 g (45). It is possible that those with exercise-induced GI symptoms experience a lowering of their FODMAP thresholds due to physiological changes that occur during exercise (19).

This study utilized self-reports of symptoms over time in order to obtain a general picture of symptom frequency. This limits the conclusions that were able to be drawn when looking at relationships to diet and exercise behaviors. More recent intervention studies of athlete GI symptoms in relation to diet and exercise use symptom severity scales immediately during and after exercise. While this acute measurement allows for more direct relationships to be seen, the intent of this study was more exploratory in nature and intended to bring awareness to the influence of FODMAPs in sports nutrition products in addition to intake from habitual diets.

Additionally, given the large number of statistical tests conducted in this study, particularly on the habitual FODMAP data, type 1 error is possible. As previously stated, associations were intended to be thought-provoking explorations of potential links between habitual intake of various FODMAPs and GI symptoms and not directly causal relationships. The study also demonstrates how dietary FODMAP analysis tools, such as the CNAQ, may be used to explore individual athlete diets to find targets for FODMAP reduction and potential symptom mitigation. It should be noted that, while false positive findings may have occurred, when significant differences in median FODMAP intakes were found between individual symptom presence categories, higher median intakes were seen among athletes with the symptoms present compared to not present with the exception of sorbitol and constipation during training.

The classification of potentially high FODMAP foods included general free response entries such as “fruit,” “veggies,” or “carbs.” While it is possible that athletes are consuming high FODMAP foods within these broad categories, this categorization may have resulted in an overestimation. Nonetheless, the categorizations were not used to quantitatively compare to GI symptoms and even the more strictly defined specific high FODMAP foods were consumed by over 60% of athletes.

All data in this work was collected via online questionnaires, which has inherent limitations as discussed in previous work with the EAQ (37). Of the 234 athletes who qualified to participate, 80 completed the CNAQ and it is unclear what factors led to final participation. The demographics of the athletes who completed the CNAQ are similar to those who completed the EAQ, although the proportion of athletes with diagnosed or undiagnosed IBS was greater among those completing the CNAQ (17.8% compared to 9.8%). Due to the nature of the analyses using the CNAQ data, the total sample size of 73 athletes was often divided into two groups (high or low FODMAP and with or without symptoms). While a larger sample size may find additional relationships between FODMAPs and symptoms, there are many reasons why such associations may not appear, mainly due to the individual susceptibilities and thresholds mentioned previously. A linear relationship between intakes and symptoms would not be expected and it is possible that those with high intakes of certain FODMAPs have found that they can safely consume such amounts. Additionally, it was necessary for athletes completing the CNAQ to have also previously completed the EAQ, and that limited the pool of potential athletes. The CNAQ was also validated for use in a healthy, Australian population and not U.S. endurance athletes. The gender and age demographics are similar between the CNAQ validation population and this study; however, the participating endurance athletes had higher incomes and potentially more education, although differences in demographic collection made direct comparisons difficult. It should be noted that in the time since this study, a new FODMAP calculator has been developed to assess FODMAP content of diets for intervention studies (https://monashfodmapcalculator.com.au/#page-top). This would provide a more accurate assessment of daily FODMAP intake as opposed to the retrospective food frequency questionnaire type measurement of the CNAQ which relies on an athlete's food knowledge and memory.

### Practical Applications

The questionnaires (EAQ and CNAQ), in conjunction with the FODMAP analysis data, can be utilized by sports dietitians working with symptomatic athletes in order to identify target areas within athletes' diets where consumption of FODMAP is particularly high or specific FODMAP types that are consumed at high levels. This approach exploits the individualized nature of FODMAP sensitivities discussed previously and could be a preliminary approach to FODMAP reduction by athletes. Traditionally, a low FODMAP diet intervention involves a strict exclusion diet for a period of at least 4 weeks, followed by gradual re-challenge or reintroduction of small amounts of different FODMAP groups (17, 76). This restrictive period may be difficult for athletes who want to continue training during this time if calories and other nutrients are not sufficiently replaced. Alternatively, athletes could first try the so-called bottom-up approach (62), which involves cutting back on certain FODMAPs that they are consuming at high levels. This could be achieved by focusing on habitual foods in addition to changing nutrition surrounding exercise to lower FODMAP options. A second approach that may be more applicable to this population involves the acute elimination of high FODMAP foods during a period of 24–72 h before key strenuous workouts or competitions (36). This would reduce the fermentable carbohydrate load during periods of high stress and may help decrease symptoms at these time points. The use of any of these methods highlights the importance of consulting a trained dietitian to ensure proper fueling while potentially reducing detrimental symptoms. Athletes who already have experience with FODMAPs and know their specific sensitivities can utilize the sports nutrition product information when selecting products for training and competition. Caution should be taken when selecting products not previously analyzed for FODMAP content, since ingredient lists are not always accurately predictive of FODMAP content (35).

## Conclusions

While this study highlighted some of the relationships between nutrition product types or FODMAP intakes and lower GI symptoms, the key finding was the overall high FODMAP intake levels by endurance athletes, both habitually and surrounding exercise. This was emphasized by the high FODMAP pre-race food choices and the high levels found in many popular sports nutrition products. This is particularly true of the sports nutrition products when multiple servings are consumed in a short period of time, as in a training or competition situation when the recommended carbohydrate intake is 30–90 g/h (23). The FODMAPs in these products are added on to high habitual levels reported on the CNAQ. The findings in this study provide further evidence for the potential of low FODMAP interventions to help mitigate GI symptoms of endurance athletes, with targets of everyday diet, pre-race nutrition, and sports nutrition products.

## Data Availability Statement

The raw data supporting the conclusions of this article will be made available by the authors, without undue reservation.

## Ethics Statement

The studies involving human participants were reviewed and approved by University of Illinois Institutional Review Board. The ethics committee waived the requirement of written informed consent for participation.

## Author Contributions

Study was designed by LK and S-YL, in consultation with JM, JB, and NB. Data were collected and analyzed by LK with interpretation by LK, S-YL, JM, JB, and NB. Manuscript preparation and revision was performed by LK, S-YL, JM, JB, and NB. All authors contributed to the article and approved the submitted version.

## Conflict of Interest

JM works in a department that financially benefits from the sales of a digital application and booklets on the low FODMAP diet. Funds raised contribute to research of the Department of Gastroenterology and to the University. The remaining authors declare that the research was conducted in the absence of any commercial or financial relationships that could be construed as a potential conflict of interest. The reviewer SS declared a past collaboration with one of the authors JM to the handling editor.
