# Supplementary material for: High Fermentable Oligosaccharides, Disaccharides, Monosaccharides, and Polyols (FODMAP) Consumption Among Endurance Athletes and Relationship to Gastrointestinal Symptoms
Source: Front Nutr. 2021 Apr 20;8:637160. doi: 10.3389/fnut.2021.637160 (PMC8095397; doi:10.3389/fnut.2021.637160)
Supplement: Supplementary file 1 [file Data_Sheet_1.pdf]

Supplementary Table 1. Sports nutrition products analyzed for fermentable oligosaccharides, disaccharides, monosaccharides, and polyols (FODMAP) content with specific flavor and product ingredients. Sports drink/thirst quenchers are abbreviated with Bev (for beverage), solids with either Bar or Waf (for waffle), and gel/gummies with either Gel or Gum (for gummy).

Products	Flavor	Ingredients
Sports Drink/ Thirst Quencher		
Bev1	lemon-lime	water, sugar, dextrose, citric acid, salt, sodium citrate, monopotassium phosphate, gum arabic, glycerol ester of rosin, natural flavor, yellow 5
Bev2	berry	water, high fructose corn syrup, less than 0.5% of: citric acid, salt and magnesium chloride and calcium chloride and mono-potassium phosphate (electrolyte sources), natural flavors, modified food starch, calcium disodium EDTA (to protest color), medium chain triglycerides, sucrose acetate isobutyrate, vitamin B3 (niacinamide), vitamin B6 (pyridoxine hydrochloride), vitamin B12, Blue 1
Bev3	lemon-lime	sodium (bicarbonate and carbonate), potassium (bicarbonate), magnesium (sulfate), calcium (carbonate), vitamin c (as ascorbic acid), citric acid, dextrose (d-glucose), natural flavors, monk fruit extract, stevia leaf extract, avocado oil, riboflavin (for color)
Bev4	unflavored	dextrose (glucose), sucrose, citric acid, sodium citrate, sea salt, organic flavor, potassium chloride, magnesium citrate, calcium carbonate
Bev5	lemon-lime	cane sugar, dextrose, sodium citrate, citric acid, magnesium lactate, calcium citrate, potassium citrate, lemon oil, lime oil, lemon juice, lime juice, ascorbic acid (vitamin C)
Solid		
Bar1	white chocolate macadamia nut	organic brown rice syrup, organic rolled oats, soy protein isolate, organic cane syrup, organic roasted soybeans, rice flour, macadamia nuts, organic cane sugar, organic oat fiber, organic soy flour, cocoa butter, organic high oleic sunflower oil, natural flavors, organic cocoa butter, sea salt, soy flour, barley malt extract, soy lecithin, mixed tocopherols (antioxidant), dicalcium phosphate, magnesium oxide, ascorbic acid (vit C), DL-alpha tocopheryl acetate (vit E), beta carotene (vit A), niacinamide (vit B3), ergocalciferol (vit D2), thiamine mononitrate (vit B1), pyridoxine hydrochloride (vit B6), riboflavin (vit B2), cyanocobalamin (vit B12)
Waf1	honey	organic wheat flour, organic palm fruit oil, organic rice syrup, organic can sugar, organic honey, organic whole wheat flour, organic soy flour, sea salt, soy lecithin, organic spices, baking soda

Supplementary Table 1 cont.

Products	Flavor	Ingredients
Bar2	salted caramel	brown rice syrup, gluten-free oats, cashew butter, brown rice flour, honey, salted caramel toffee pieces (cane sugar, butter, sea salt, soy lecithin, citric acid), peanuts, rice protein, crisp rice (rice flour, sugar, salt, calcium carbonate), flaxseeds, natural flavors, sea salt
Waf2	salted caramel	gluten free flour blend (organic rice flour, organic potato starch, organic tapioca flour), organic palm fruit oil, organic brown rice syrup, organic cane sugar, organic eggs, organic soy flour, sea salt, organic honey, natural flavor, xanthan gum, baking soda, soy lecithin
Gel/Gummies		
Gel1	vanilla	maltodextrin, water, fructose, l-leucine, potassium citrate, sodium citrate, citric acid, calcium carbonate, l-valine, sea salt, natural flavor, green tea (leaf) extract (contains caffeine), gellan gum, l-isoleucine, sunflower oil, sodium benzoate (preservative), potassium sorbate (preservative)
Gel2A	honey	honey, water, potassium citrate, salt, niacinamide (vit B3), calcium pantothenate (vit B5), pyridoxine hydrochloride (vit B6), riboflavin (vit B2), thiamine monocitrate (vit B1), cyanocobalamin (vit B12)
Gel2B	vanilla	organic tapioca syrup, organic honey, water, potassium citrate, organic vanilla flavor, citric acid, salt
Gel3A	vanilla	maltodextrin, water, energy smart ® (grape juice, rice dextrin), vanilla extract, cultured dextrose, citric acid, potassium chloride, salt, l-leucine, l-alanine, l-valine, l-isoleucine
Gel3B	apple cinnamon	maltodextrin, water, apple juice concentrate, energy smart ® (grape juice, rice dextrin), cultured dextrose, cinnamon, ascorbic acid, vanilla extract, malic acid, potassium carbonate, salt, l-leucine, l-alanine, l-valine, l-isoleucine
Gel4	vanilla	organic maltodextrin, organic dried cane syrup, water, natural flavor, sea salt, potassium citrate
Gum1	citrus	organic tapioca syrup, organic dried cane syrup, organic maltodextrin, pectin, citric acid, potassium citrate, natural flavor, sea salt, organic sunflower oil, carnauba wax

Supplementary Table 2. Nutrition product type consumed before training and gastrointestinal (GI) symptoms during training.

Nutrition	Symptom	H* (n)	Total p	Group 1 (n, mean rank)†	Group 2 (n, mean rank)†	p‡
Sports drink/thirst quencher	GI cramps/pain	13.669 (404)	.008	Never (146, 209.27)	Always (22, 118.50)	.003
				Rarely (104, 208.98)	Always (22, 118.50)	.005
				Sometimes (97, 202.57)	Always (22, 118.50)	.013
				Often (35, 207.61)	Always (22, 118.50)	.031
	Urge to defecate	14.213 (403)	.007	Never (145, 205.59)	Always (24, 129.40)	.019
				Rarely (104, 219.49)	Always (24, 129.40)	.003
				Sometimes (95, 203.34)	Always (24, 129.40)	.036
Solid food	GI cramps/pain	12.165 (410)	.016	Never (28, 162.38)	Often (113, 229.13)	.049
	Bloating	15.861 (410)	.003	Never (28, 138.14)	Sometimes (142, 213.56)	.009
				Never (28, 138.14)	Often (114, 223.63)	.002
Gel/gummy	GI cramps/pain	15.112 (402)	.004	Sometimes (72, 233.05)	Always (22, 131.34)	.001
Homemade product/ something else	Bloating	28.536 (404)	<.001	Never (160, 176.21)	Sometimes (97, 237.90)	<.001
				Never (160, 176.21)	Always (21, 271.00)	.002
				Rarely (83, 189.51)	Always (21, 271.00)	.021
	Constipation	17.492 (400)	.002	Never (159, 187.07)	Often (46, 232.03)	.027
				Rarely (82, 184.30)	Often (46, 232.03)	.038

*Kruskal-Wallis H value is also $\chi^2(4)$

† Post hoc pairwise comparisons with Dunn's procedure and Bonferroni correction comparing the product consumption frequency categories shown under Group 1 to Group 2. The n of each group indicates the number of participants responding with that nutrition product consumption frequency. Mean ranks of the Kruskal-Wallis H test are used to compare the effect of usage frequency on specific GI symptoms, with larger mean ranks indicating more frequent symptoms.

‡ *p*-value for individual pairwise comparisons

Supplementary Table 3. Nutrition product type consumed before training and gastrointestinal (GI) symptoms within 2hrs after training.

Nutrition	Symptom	H* (n)	Total p	Group 1 (n, mean rank)†	Group 2 (n, mean rank)†	p‡
Sports drink/thirst quencher	Flatulence	13.714 (403)	.008	Never (145, 178.85)	Rarely (104, 225.96)	.010
Sports drink/energy drink	Diarrhea	11.278 (407)	.024	Never (208, 193.28)	Rarely (105, 234.34)	.017
	Constipation	13.439 (404)	.009	Never (207, 194.35)	Rarely (104, 227.00)	.025
Gel/gummy	Bloating	10.528 (403)	.032	Never (160, 186.01)	Sometimes (71, 230.11)	.038
	Flatulence	9.983 (401)	.041	Never (159, 186.66)	Sometimes (72, 235.93)	.017
Homemade product/ something else	GI cramps/pain	12.916 (404)	.012	Never (161, 181.80)	Always (21, 254.19)	.046

*Kruskal-Wallis H value is also $\chi^2(4)$

† Post hoc pairwise comparisons with Dunn's procedure and Bonferroni correction comparing the product consumption frequency categories shown under Group 1 to Group 2. The n of each group indicates the number of participants responding with that nutrition product consumption frequency. Mean ranks of the Kruskal-Wallis H test are used to compare the effect of usage frequency on specific GI symptoms, with larger mean ranks indicating more frequent symptoms.

‡ *p*-value for individual pairwise comparisons

Supplementary Table 4. Nutrition product type consumed during training and gastrointestinal (GI) symptoms during training.

Nutrition	Symptom	H* (n)	Total p	Group 1 (n, mean rank)†	Group 2 (n, mean rank)†	p‡
Solid food	GI cramps/pain	15.065 (406)	.005	Never (102, 198.82)	Always (19, 115.58)	.027
				Rarely (84, 210.44)	Always (19, 115.58)	.008
				Sometimes (134, 204.04)	Always (19, 115.58)	.012
				Often (67, 225.78)	Always (19, 115.58)	.001
	Constipation	10.757 (402)	.029	Never (101, 180.07)	Often (66, 223.81)	.020
Gel/gummy	Bloating	10.314 (407)	.035	Never (47, 161.66)	Often (125, 220.30)	.017
	Urge to defecate	10.760 (406)	.029	Never (47, 158.13)	Sometimes (130, 219.82)	.012
Homemade product/ something else	Bloating	14.850 (403)	.005	Never (197, 182.67)	Rarely (75, 232.16)	.008
	Constipation	23.898 (399)	<.001	Never (195, 178.76)	Rarely (75, 228.76)	<.001
				Never (195, 178.76)	Sometimes (80, 215.11)	.020

*Kruskal-Wallis H value is also $\chi^2(4)$

† Post hoc pairwise comparisons with Dunn's procedure and Bonferroni correction comparing the product consumption frequency categories shown under Group 1 to Group 2. The n of each group indicates the number of participants responding with that nutrition product consumption frequency. Mean ranks of the Kruskal-Wallis H test are used to compare the effect of usage frequency on specific GI symptoms, with larger mean ranks indicating more frequent symptoms.

‡ *p*-value for individual pairwise comparisons

Supplementary Table 5. Nutrition product type consumed during training and gastrointestinal (GI) symptoms within 2hrs after training.

Nutrition	Symptom	H* (n)	Total p	Group 1 (n, mean rank)†	Group 2 (n, mean rank)†	p‡
Gel/gummy	Bloating	10.179 (408)	.038	Never (47, 158.86)	Sometimes (131, 213.67)	.029
	Flatulence	12.978 (406)	.011	Never (46, 148.54)	Sometimes (131, 211.83)	.010
				Never (46, 148.54)	Often (125, 209.64)	.015
				Never (46, 148.54)	Always (49, 217.44)	.027
	Urge to defecate	12.092 (407)	.017	Never (47, 150.62)	Sometimes (131, 211.42)	.016
				Never (47, 150.62)	Often (126, 209.57)	.024
				Never (47, 150.62)	Always (48, 218.57)	.035
	Defecation	13.888 (407)	.008	Never (47, 147.06)	Sometimes (131, 210.47)	.010
				Never (47, 150.62)	Often (125, 215.41)	.004
				Never (47, 150.62)	Always (49, 213.59)	.041
Homemade product/ something else	GI cramps/pain	11.749 (403)	.019	Never (198, 186.73)	Rarely (74, 231.59)	.027
	Bloating	10.010 (403)	.040	Never (198, 188.59)	Rarely (75, 230.13)	.040
	Constipation	15.795 (401)	.003	Never (197, 186.95)	Rarely (75, 233.43)	.001

*Kruskal-Wallis H value is also $\chi^2(4)$

† Post hoc pairwise comparisons with Dunn's procedure and Bonferroni correction comparing the product consumption frequency categories shown under Group 1 to Group 2. The n of each group indicates the number of participants responding with that nutrition product consumption frequency. Mean ranks of the Kruskal-Wallis H test are used to compare the effect of usage frequency on specific GI symptoms, with larger mean ranks indicating more frequent symptoms.

‡ *p*-value for individual pairwise comparisons

Supplementary Table 6. Nutrition product type consumed before competition and gastrointestinal (GI) symptoms during competition.

Nutrition	Symptom	H* (n)	Total p	Group 1 (n, mean rank)†	Group 2 (n, mean rank)†	p‡
Sports drink/ energy drink	Bloating	14.297 (398)	.006	Never (201, 180.75)	Rarely (73, 227.54)	.012
	Constipation	12.466 (393)	.014	Never (200, 189.37)	Rarely (71, 227.02)	.007
Solid food	Constipation	10.687 (399)	.030	Sometimes (79, 225.08)	Always (138, 187.77)	.012
Homemade product/ something else	GI cramps/pain	15.024 (400)	.005	Never (182, 181.28)	Sometimes (75, 233.51)	.005
	Bloating	16.295 (400)	.003	Never (182, 178.50)	Sometimes (75, 232.05)	.002
	Defecation	13.148 (395)	.011	Never (180, 178.26)	Sometimes (74, 221.95)	.024

*Kruskal-Wallis H value is also $\chi^2(4)$

† Post hoc pairwise comparisons with Dunn's procedure and Bonferroni correction comparing the product consumption frequency categories shown under Group 1 to Group 2. The n of each group indicates the number of participants responding with that nutrition product consumption frequency. Mean ranks of the Kruskal-Wallis H test are used to compare the effect of usage frequency on specific GI symptoms, with larger mean ranks indicating more frequent symptoms.

‡ *p*-value for individual pairwise comparisons

Supplementary Table 7. Nutrition product type consumed before competition and gastrointestinal (GI) symptoms within 2hrs after competition.

Nutrition	Symptom	H* (n)	Total p	Group 1 (n, mean rank)†	Group 2 (n, mean rank)†	p‡
Sports drink/energy drink	Flatulence	12.254 (399)	.016	Never (200, 183.86)	Always (28, 247.50)	.042
Solid food	Urge to defecate	12.870 (401)	.012	Never (33, 145.61)	Sometimes (80, 226.10)	.012
	Defecation	22.409 (403)	<.001	Never (33, 125.95)	Rarely (59, 201.07)	.020
				Never (33, 125.95)	Sometimes (79, 232.31)	<.001
				Never (33, 125.95)	Often (93, 192.67)	.032
				Never (33, 125.95)	Always (139, 209.47)	.001
Gel/gummy	Bloating	16.457 (398)	.002	Never (136, 175.89)	Rarely (70, 231.68)	.003
Homemade product/ something else	GI cramps/pain	20.074 (400)	<.001	Never (181, 175.22)	Sometimes (75, 232.91)	.001
	Bloating	15.267 (399)	.004	Never (181, 177.96)	Sometimes (75, 220.35)	.037
	Constipation	12.353 (395)	.015	Never (179, 183.44)	Sometimes (74, 219.35)	.029

*Kruskal-Wallis H value is also $\chi^2(4)$

† Post hoc pairwise comparisons with Dunn's procedure and Bonferroni correction comparing the product consumption frequency categories shown under Group 1 to Group 2. The n of each group indicates the number of participants responding with that nutrition product consumption frequency. Mean ranks of the Kruskal-Wallis H test are used to compare the effect of usage frequency on specific GI symptoms, with larger mean ranks indicating more frequent symptoms.

‡ *p*-value for individual pairwise comparisons

Supplementary Table 8. Nutrition product type consumed during competition and gastrointestinal (GI) symptoms during competition.

Nutrition	Symptom	H* (n)	Total p	Group 1 (n, mean rank)†	Group 2 (n, mean rank)†	p‡
Solid food	Bloating	10.199 (398)	.037	Never (104, 175.08)	Sometimes (120, 216.57)	.034
Gel/gummy	Urge to defecate	10.811 (408)	.029	Never (37, 153.93)	Sometimes (53, 207.70)	.045
				Never (37, 153.93)	Often (142, 215.18)	.031
	Bloating	9.676 (412)	.046	Never (36, 154.54)	Sometimes (140, 216.15)	.024
				Never (36, 154.54)	Often (156, 198.20)	.048
Homemade product/ something else	Bloating	13.729 (396)	.008	Never (209, 182.84)	Rarely (69, 229.10)	.015

*Kruskal-Wallis H value is also $\chi^2(4)$

† Post hoc pairwise comparisons with Dunn's procedure and Bonferroni correction comparing the product consumption frequency categories shown under Group 1 to Group 2. The n of each group indicates the number of participants responding with that nutrition product consumption frequency. Mean ranks of the Kruskal-Wallis H test are used to compare the effect of usage frequency on specific GI symptoms, with larger mean ranks indicating more frequent symptoms.

‡ *p*-value for individual pairwise comparisons

Supplementary Table 9. Nutrition product type consumed during competition and gastrointestinal (GI) symptoms within 2hrs after competition.

**Nutrition**	**Symptom**	**H* (n)**	**Total p**	**Group 1 (n, mean rank)†**	**Group 2 (n, mean rank)†**	**p‡**
Homemade product/ something else	GI cramps/pain	15.154 (397)	.004	Never (209, 182.44)	Rarely (69, 230.93)	.013

*Kruskal-Wallis H value is also $\chi^2(4)$

† Post hoc pairwise comparisons with Dunn's procedure and Bonferroni correction comparing the product consumption frequency categories shown under Group 1 to Group 2. The n of each group indicates the number of participants responding with that nutrition product consumption frequency. Mean ranks of the Kruskal-Wallis H test are used to compare the effect of usage frequency on specific GI symptoms, with larger mean ranks indicating more frequent symptoms.

‡ *p*-value for individual pairwise comparisons

Supplementary Table 10. Average daily nutritional intake of endurance athletes (n=73).

Nutrient	Daily intake (Mean±SD)
Energy	2157.0±778.6
Carbohydrate	233.8±97.9
Fat	89.9±41.2
Protein	99.1±37.5
Fiber	36.4±19.1
